# S68. SYMPTOMS, NEUROCOGNITION, SOCIAL COGNITION AND METACOGNITION IN SCHIZOPHRENIA: A NETWORK ANALYSIS

**DOI:** 10.1093/schbul/sby018.855

**Published:** 2018-04-01

**Authors:** Ilanit Hasson-Ohayon, Gil Goldzweig, Adi Lavie, Lauren Luther, Paul Lysaker

**Affiliations:** 1 Bar-Ilan University; 2 School of Behavioral Sciences, The Academic College of Tel-Aviv; 3 Roudebush VA Medical Center and the Indiana University School of Medicine

## Abstract

**Background:**

Schizophrenia is associated with broad range of phenomena which affect function and represent significant barriers to recovery. These include semi-independent forms of psychopathology, disturbances in neurocognition, social cognition and metacognition. The current study explores the paths through which these constructs affect each other and whether some of these phenomena play a relatively more or less central role than others as they interact. Answers to these questions seem essential to choosing which of a dizzying array of problems should be targeted by treatment.

**Methods:**

Data was collected from 81 adult outpatients with schizophrenia or schizoaffective disorder, recruited at a Veterans’ Affairs Medical Center and a community mental health center in Indiana, USA. Network analysis which explored the relative relationships of five groups of symptoms (positive, negative, disorganization, hostility and emotional discomfort), six domains of neurocognition, four domains of social cognition and four domains of metacognition with one another was conducted. The analysis produces the following centrality measures: 1) strength of items within a network according to their sum weighted connections; 2) closeness between items that reflect the distance from a particular item to all others; 3) betweenness which reflect the number of times that an item appears on the shortest path between two other items.

**Results:**

A clear differentiation between metacognition, social cognition, neurocognition and symptoms was observed. The only outliers were social cognition attribution, which was close to the symptoms area, and the cognitive symptoms factor that was found close to the neuro-cognition area. The social cognition was found in an “intermediate” area between the metacognition and neurocognition. Metacognition variables were the closest to the symptoms variables. The strongest nodes are: metacognition-self reflectivity, theory of mind measures of social cognition and visual memory. The nodes with the highest closeness measure were self-reflectivity sub-scale of metacognition and theory of mind of social cognition. The node with the highest betweenness measure was metacognition self-reflectivity.

**Discussion:**

The centrality of the self-experience in schizophrenia is emphasized in phenomenological, theoretical as well as empirical literature and can be traced back to earlier writing on schizophrenia. Accordingly, a sense of barren or diminished self, problems in self-reflection and self-clarity as well as difficulties in agency and ownership over one’s thoughts, feelings and sensations which is necessary for creating meaning were reported and discussed. The current study adds to this body of literature the finding that in a network which includes symptoms, social cognition, neuro cognition and metacognition variables, self-reflection is standing out as being a central connector that has the strongest relationship with other variables. As such it impacts all the network, and interventions targeting metacognitive self-reflection are expected to have secondary effects on additional constructs in the network- i.e additional elements of metacognition, social cognition, neurocognition and symptoms.

